# Human RPE Stem Cells Grown into Polarized RPE Monolayers on a Polyester Matrix Are Maintained after Grafting into Rabbit Subretinal Space

**DOI:** 10.1016/j.stemcr.2013.11.005

**Published:** 2014-01-02

**Authors:** Boris V. Stanzel, Zengping Liu, Sudawadee Somboonthanakij, Warapat Wongsawad, Ralf Brinken, Nicole Eter, Barbara Corneo, Frank G. Holz, Sally Temple, Jeffrey H. Stern, Timothy A. Blenkinsop

**Affiliations:** 1 Department of Ophthalmology, University of Bonn, Bonn 53127, Germany; 2 Mettapracharak Eye Institute, Raikhing, Nakhon Pathom 73210, Thailand; 3 Department of Ophthalmology, University of Muenster, Muenster 48149, Germany; 4 Neural Stem Cell Institute, Rensselaer, NY 12144, USA

## Abstract

Transplantation of the retinal pigment epithelium (RPE) is being developed as a cell-replacement therapy for age-related macular degeneration. Human embryonic stem cell (hESC) and induced pluripotent stem cell (iPSC)-derived RPE are currently translating toward clinic. We introduce the adult human RPE stem cell (hRPESC) as an alternative RPE source. Polarized monolayers of adult hRPESC-derived RPE grown on polyester (PET) membranes had near-native characteristics. Trephined pieces of RPE monolayers on PET were transplanted subretinally in the rabbit, a large-eyed animal model. After 4 days, retinal edema was observed above the implant, detected by spectral domain optical coherence tomography (SD-OCT) and fundoscopy. At 1 week, retinal atrophy overlying the fetal or adult transplant was observed, remaining stable thereafter. Histology obtained 4 weeks after implantation confirmed a continuous polarized human RPE monolayer on PET. Taken together, the xeno-RPE survived with retained characteristics in the subretinal space. These experiments support that adult hRPESC-derived RPE are a potential source for transplantation therapies.

## Highlights

• Adult hRPESC-derived RPE had comparable in vitro characteristics to fetal hRPE
• hRPE monolayers survived 4 weeks on PET carriers under the rabbit retina
• Better xenograft survival may be due to the maintained hRPE cell polarity
• Atrophy of the retina overlaying the hRPE xenograft remains a future challenge

## Graphical Abstract

**Figure:**
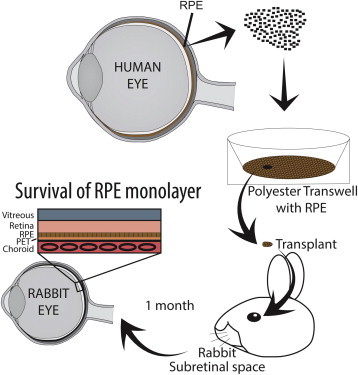

## Teaser

Stanzel et al. have transplanted polarized monolayers of human retinal pigment epithelium (hRPE) grown on polyester membranes derived from adult hRPE stem cells or fetal tissue under the rabbit retina. The xeno-RPE survived 4 weeks with retained cell polarity characteristics in the subretinal space. These experiments support that adult hRPESC-derived RPE are a potential source for transplantation therapies.

## Introduction

The retinal pigment epithelium (RPE) is a cellular monolayer between the retina and the underlying choroidal vasculature. The RPE participates actively in the visual process, notably by supporting the diurnal replenishment of the photoreceptors (Strauss, 2005). RPE dysfunction significantly contributes to the pathophysiology of age-related macular degeneration (AMD), a leading cause of blindness (Lim et al., 2012). There are currently no disease-altering therapies available for the vast majority (over 85%) of AMD patients that suffer from the dry form of the disease, which is characterized by extracellular deposits termed drusen beneath the RPE and subsequent RPE atrophy in the macula. The remaining approximately 15% of patients have wet AMD, in which neovascularization invades from the choroid; for these patients, repeated intravitreal injections with antiangiogenic drugs offer a highly effective, albeit palliative, treatment (Singer et al., 2012). Replacement of dysfunctional submacular RPE with a cell-based therapeutic agent represents a potentially curative treatment strategy (Binder et al., 2007). Some previous attempts in patients have been shown to improve vision, but most were limited by immune reactions, surgical complications, late-stage disease, or lack of an adequate RPE cell source (Stanzel and Holz, 2012). Translocation of an autologous patch of RPE/choroid remains clinically the most popular approach, because some patients benefit from the procedure, despite its high complication rates (van Zeeburg et al., 2012).

With the development of RPE differentiation protocols from human embryonic stem cells (hESCs) and induced pluripotent stem cells (iPSCs) (Hirami et al., 2009; Klimanskaya et al., 2004), RPE transplantation has experienced a powerful renaissance, as scientists and clinicians envision an unlimited supply of RPE for transplantation. However, much is still not understood with regard to the physiology of stem-cell-derived RPE (Liao et al., 2010) and transplantation into patients is in the early stages. Pilot data from a phase I/II trial (NCT01226628 and NCT01344993) with a suspension of hESC-derived RPE injected in patients with dry AMD or Stargardt’s disease suggest a favorable safety profile and some limited improvement in vision (Schwartz et al., 2012); further dose-escalation in this multicenter study is on-going. This is encouraging, given that prior studies using RPE cell suspensions showed they failed to survive or function on aged submacular Bruch’s membrane (Sugino et al., 2011) and are more likely to be rejected than are RPE monolayers (Diniz et al., 2013).

A cultured human RPE monolayer that exhibits the physiology of its native counterpart could be a valuable alternative to an RPE-cell suspension. This type of culture has been readily attained using fetal- or pluripotent-stem-cell-derived RPE. However, establishing such cultures from adult RPE has proven difficult and inconsistent, due to its propensity to undergo epithelial-mesenchymal transition (reviewed in Burke, 2008). We have optimized culture conditions that robustly activate a subpopulation of adult human RPE stem cells (RPESC), expand, and then differentiate them into highly pure RPE monolayers that exhibit physiological features of native RPE (Blenkinsop et al., 2013; Salero et al., 2012). This protocol allows us to explore the potential of adult RPESC-derived RPE for cell-replacement therapy. To date, we do not know which cell source will turn out to be therapeutically successful, and therefore, testing all potential candidates is important. Using a cell source derived from the adult human RPE may possess several potential advantages, such as fewer ethical concerns compared to hESC and fetal human RPE (hRPE), the possibility of routine histocompatibility leukocyte antigen matching or even autologous transplantation (using a patient’s own remaining healthy RPESCs) to minimize immunosuppression, reduced proliferative potential than hESCs or human iPSCs and therefore reduced tumorigenesis risk, and reduced threat of generating abnormal cell types.

RPE monolayers grown on cell carriers would facilitate surgical handling and long-term functionality by substituting some or all of the functions of the aged Bruch’s membrane (Binder et al., 2007). Coimplantation of differentiated RPE monolayers on a substrate has been attempted in animal models only in a few instances and with limited success (Bhatt et al., 1994; Diniz et al., 2013; Nicolini et al., 2000). Improvements would involve employing a biocompatible matrix that exhibits minimal deformation after transplantation, longer-term assessment postsurgery, and use of a large-eyed animal model for better assessment of surgical technique.

We have previously reported on a method and instrumentation to deliver ultrathin rigid-elastic cell carriers (polyester [PET]) into the subretinal space (SRS) of rabbits (Stanzel et al., 2012). Here, we demonstrate this technology can be used to deliver monolayers of human RPE on permeable polyester carriers into the SRS of the rabbit. Notably, we find RPE isolated from adult cadaver donors can expand 20-fold and survive as a polarized RPE monolayer for 1 month after transplantation, therefore representing a clinically relevant RPE cell source. Transplants were followed with state-of-the-art ophthalmic imaging technology, including spectral domain optical coherence tomography (SD-OCT), confocal scanning-laser ophthalmoscopy (cSLO), color funduscopic photography, and histology. In addition, the influence of local and systemic immunosuppression on retinal tissue alterations following xenografting was evaluated.

## Results

### Preparation and Characterization of the RPE-Carrier Monolayers

**Figure 1:**
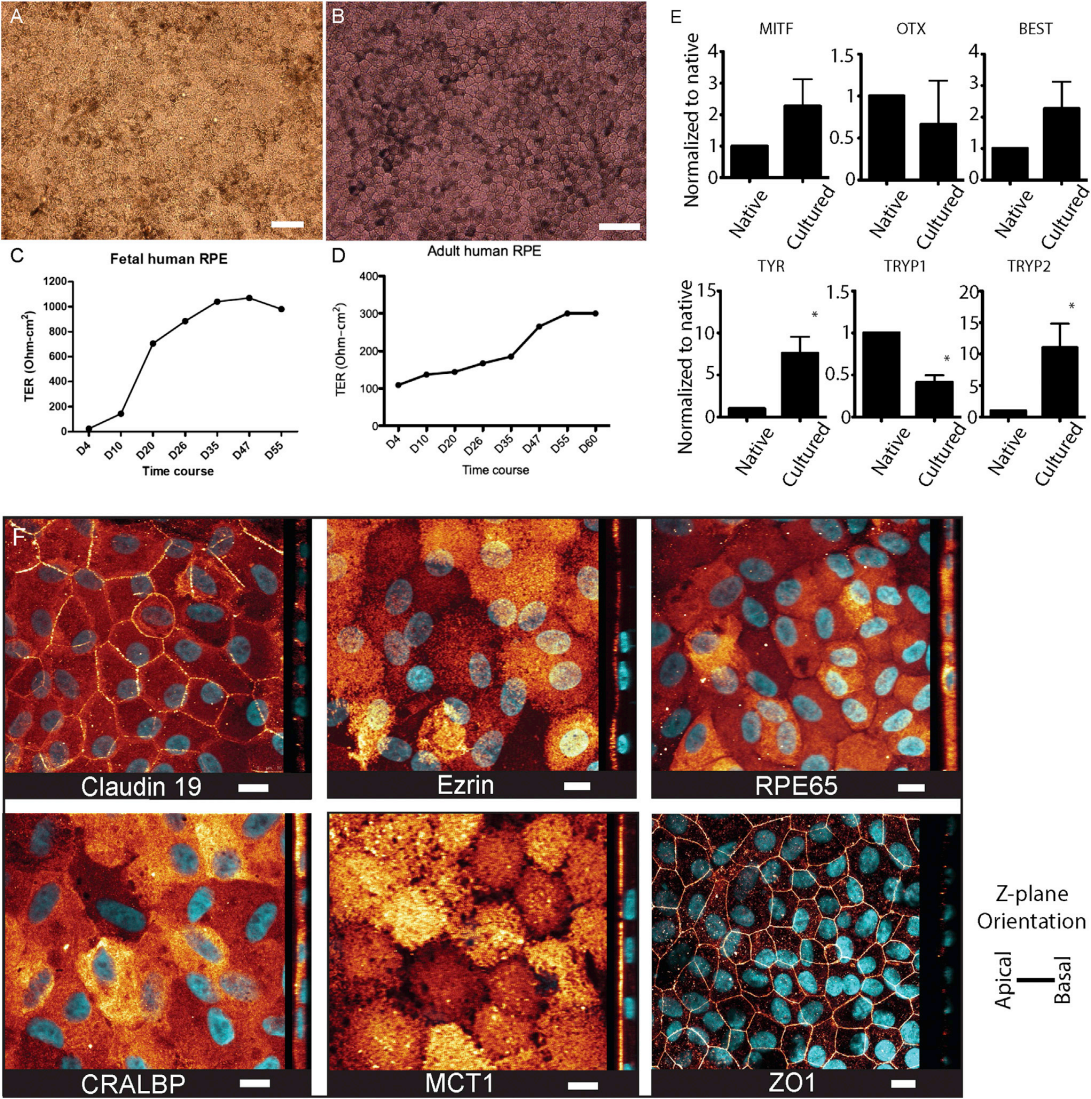
Fetal and Adult Human RPE Cultured over 2 Months Exhibit Similarities to Native RPE (A and B) Cultured (A) fetal and (B) adult hRPE display hexagonal morphology and varied pigmentation. The scale bars represent 50 μm. (C and D) TER measurements of fetal and adult hRPE, respectively. (E) mRNA expression of typical RPE transcripts was compared to its genetically matched native RPE counterpart from five adult hRPE donors, including those used for transplantation. The asterisk denotes P value > 0.01/paired t test. The error bars represent SEM. (F) Adult hRPE cultures display expression of markers typical of native RPE in their polarized localization. DAPI is cyan, whereas all other immunofluorescence is gold. Claudin 19, ezrin, ZO1, and MCT1 are preferentially located on the apical side. RPE65 and CRALBP are cytoplasmic. The scale bars represent 10 μm. See also Figure S1.

Adult human RPESC (hRPESC)-derived RPE and fetal hRPE monolayers were compared for their growth characteristics on the carrier PET material. Fetal hRPE cells seeded at 2 × 10^5^ cells/cm^2^ on PET inserts formed uniform, hexagonal monolayers by 2 weeks postconfluence, which repigmented by 6–8 weeks (Figure 1A). Cultures were used for transplantation at 2 to 3 months postconfluence, when transepithelial electrical resistance (TER) values ranged from 737–1415 Ω^∗^cm^2^, mean 1244 ± 161 Ω^∗^cm^2^ (n = 60), and 514–776 Ω^∗^cm^2^, mean 657 ± 60 Ω^∗^cm^2^ (n = 60), from two different donor cultures (Figure 1C). Fetal RPE cultured in this manner shows morphology and gene/protein expression similar to native RPE, thereby serving as a reference for adult-stem-cell-derived RPE (Liao et al., 2010).

About 5 × 10^6^ adult hRPEs were typically isolated per donor. During the first month, the cultures went through 2–4 population doublings, thus expanding to approximately 2.5 × 10^7^. We then seeded adult hRPE onto PET inserts at 1 × 10^5^ cells/cm^2^ until they formed uniform, hexagonal monolayers 4 weeks postconfluence, expanding another 2–4 more times during this passage. As a result, after 3 months, the adult RPESCs and their progeny had expanded 20-fold, and from one donor, we obtained approximately 1 × 10^8^ RPEs (Figure 1B).

We monitored adult hRPE characteristics during the culture period by measuring TER, gene expression, and polarized protein localization immunohistochemically. After 2 months on the PET substrate, when the adult hRPE monolayers exhibited a uniform, polygonal shape, TER was measured regularly to confirm development of tight junctions (Figure 1 and Figure S1 available online) and were found to range between 210–339 Ω^∗^cm^2^ at 8 weeks after plating, with a mean of 308 ± 18.7 Ω^∗^cm^2^ (n = 12) and mean 240 ± 24.9 Ω^∗^cm^2^ (n = 12) from respective donor cultures.

Gene-expression profiling was conducted using RNA extracted from adult hRPE tissue at the time of dissection (referred to as native) versus RNA extracted from adult hRPE after 2 months on PET (Figure 1E; n = 5 donors). Cultured adult hRPE exhibited largely similar but sometimes higher expression of RPE markers compared to the native tissue.

To determine purity and polarization of the adult hRPE cultures, we performed immunostaining using characteristic RPE markers, assessed by confocal imaging (Figure 1F). Claudin-19 was present along the apical-lateral membrane along with the tight-junction complex protein ZO-1, indicating the existence of a functional epithelial barrier (Peng et al., 2011). Ezrin, a membrane-associated protein involved in cytoskeletal organization, was found preferentially in RPE microvilli (Bonilha et al., 1999). The visual cycle proteins cellular retinaldehyde-binding protein (CRALBP) and RPE65 (Bunt-Milam and Saari, 1983; Redmond et al., 1998) were localized in the cytoplasm, as expected. Monocarboxylate transporter 1 (MCT1) was present apically (Philp et al., 1998).

The appropriate localization of these proteins, combined with the gene-expression pattern and TER measurements, were similar to native RPE, demonstrating appropriate physiology of the adult hRPE monolayers to be used for transplantation.

### Cultured hRPE Xenografted into Rabbit SRS

**Figure 2:**
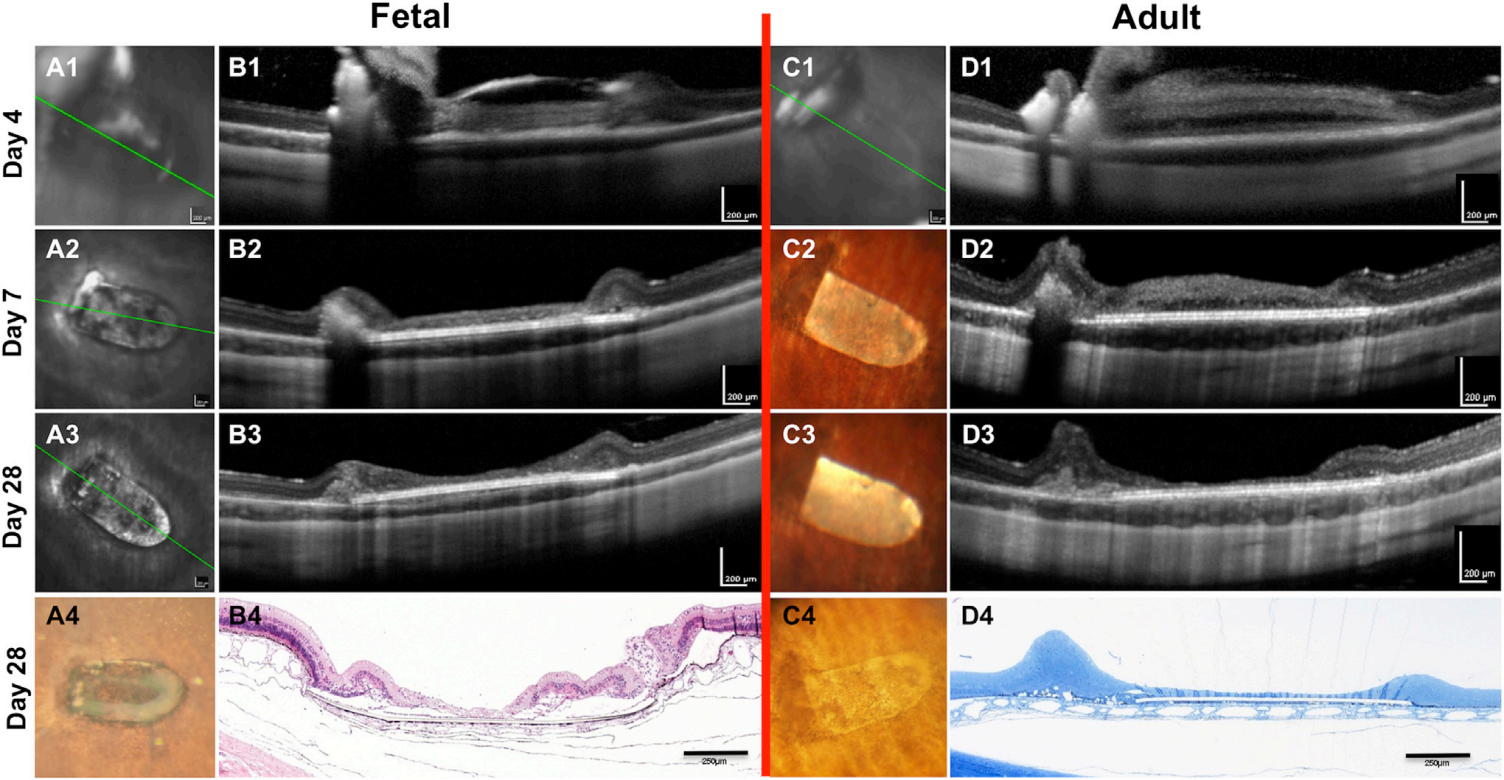
Representative Case of Fetal and hRPE Transplantation into Immune Competent-Rabbit SRS with In Vivo and Histomorphological Comparison (A and C) Funduscopy images of subretinal RPE + PET implant at respective time points, (A1–A3 and C1) infrared cSLO images, (C2 and C3) color fundus photograph, (A4 and C4) postmortem eye-cup macroscopic photograph. (B and D) Longitudinal section through center of RPE + PET implant center at indicated time points, (B1–B3 and D1–D3) SD-OCT images, and (B4 and D4) paraffin and resin histologic section, respectively. Scale bars in columns A–D and rows 1–3 are 200 μm and, in row 4, 250 μm. See also Figure S2 and S3 and Movies S1, S2, S3, and S4.

**Figure 3:**
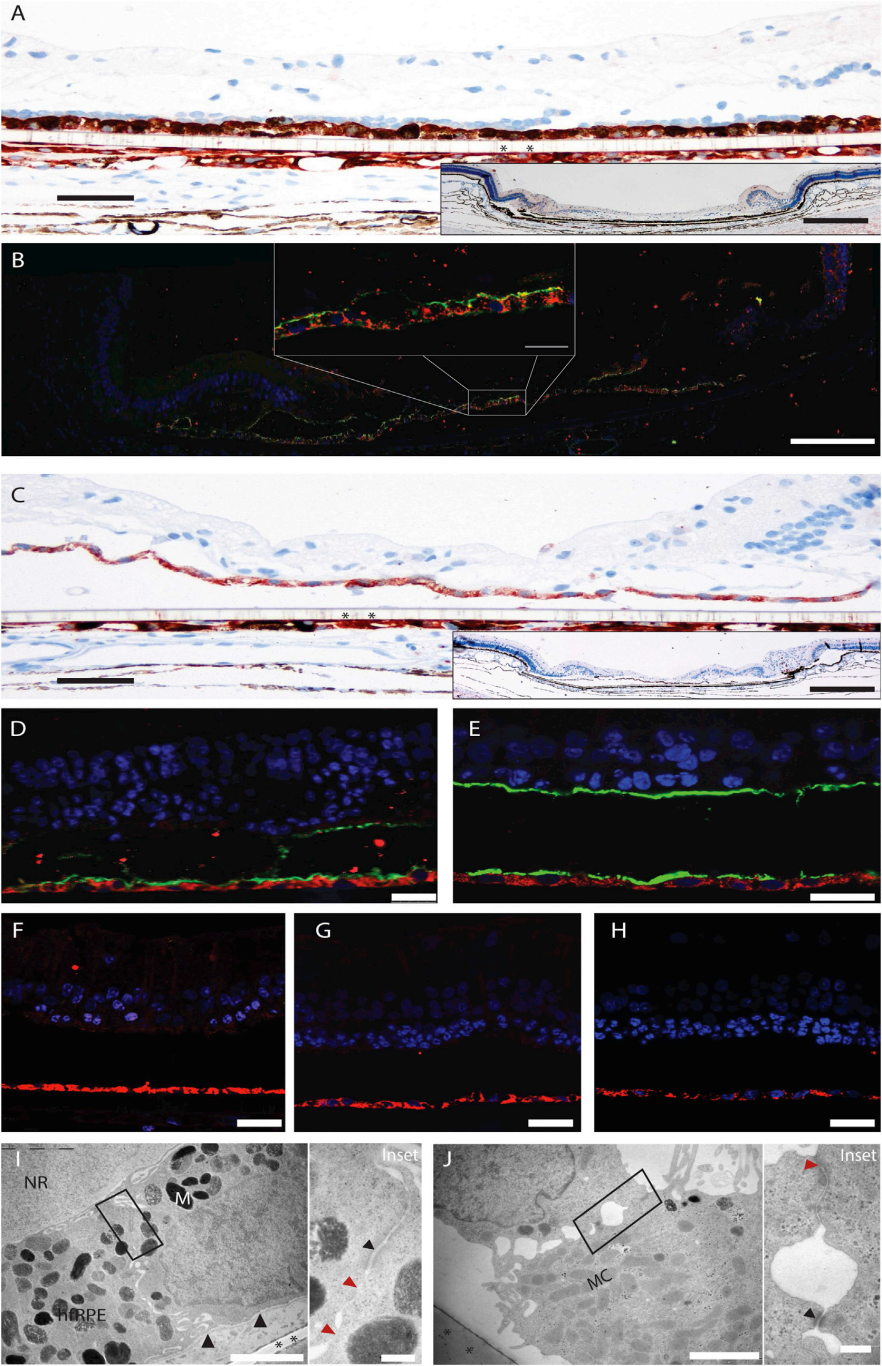
Xenografted Polarized Human RPEs Survive Subretinally for at Least 1 Month One month after hRPE transplantation, implant sites were screened for human-specific and RPE markers. (A) Fetal hRPE stained for pan-cytokeratin (scale bar, 50 μm); inset shows section overview stained with hematoxylin/eosin (scale bar, 200 μm). (B) Fetal hRPE stained for SC121 (red) and MCT1 (green; scale bars, 125 μm and 25 μm [inset]). (C) Adult hRPE stained for pan-cytokeratin (scale bar = 50 μm); inset shows section overview stained with hematoxylin/eosin (scale bar, 200 μm). (D–H) Adult hRPE stained for SC121 (red). (D) Adult hRPE stained for MCT1 (green). (E) Human adult RPE stained for ezrin (green). hRPEs transplanted into rabbit SRS show absence of expression of ki67 (F), phosphohistone H3 (G), and caspase-3 (H). Polarized fetal and adult hRPE cells were found in TEM (I and J). Nuclei with regular chromatin were found in the basal compartment, a basal lamina ([I], large black arrowhead) had formed between the xenograft and PET carrier (black asterisks). Melanosomes (M) in multiple stages, some microvilli abutting to the atrophic neural retina (NR), and junctional structures with desmosomes (small black arrowhead) and tight junctions (red arrowhead) were discerned apically. Mitochondria (MC) were seen in the basolateral part of the cell. Detachment from cell carrier (asterisk) in (J) is a histologic processing artifact. Left images in (I) and (J) taken at 10,500× magnification; right micrographs are rectangular zone in left at 25,000×; scale bars represent 2 μm/inset 0.2 μm distance in (I) and (J).

hRPE cultures grown on PET membranes were trephined to generate bullet-shaped implants approximately 1.1 mm × 2.2 mm (∼6–8000 cells), as described previously (Stanzel et al., 2012). Subretinal implantation of 45 such constructs was evaluated, which included 40 fetal and five adult human RPE monolayer implants (Table 1). The RPE monolayer transplant was placed cell-carrier down on intact host RPE within the SRS (Figures 2 and 3; Movie S1). In addition, controls with bleb retinal detachments alone (bRD) (n = 19) and PET carrier-only implants (n = 7) were performed to differentiate surgical trauma from biological effects (Figure S2; Movie S2).

On first postoperative exam (days 3–5), a white retinal opacity overlying the hRPE implant was seen on fundus photography, along with occasional triamcinolone crystals trapped around the retinotomy site. The whitish opacity was subsequently lost in both hRPE implant types, with all remaining follow-up exams (beyond 1 week postoperation [post-OP]) showing a steep edge around the implant periphery (Figure 2A for fetal and 2C for adult; Movies S3 and S4).

On the first SD-OCT follow-up (at 3–5 days post-OP), the neural retina above the fetal and adult hRPE implant showed an increased overall thickness, with loss of typical retinal reflectance layering, as well as slowed clearance of subretinal fluid from the bRD (Figure 2, B1 and D1, respectively). Xenografted adult hRPE on PET followed similar, albeit slightly delayed, patterns as seen with the fetal transplants. Notably, however, the retina was typically adherent to both hRPE implant variants on the apical surface, and the implant plus carrier appeared apposed to the host RPE layer on the basal surface. The choroidal vascular layer underneath both hRPE implant types appeared to have less reflectivity and slightly increased volume compared to adjacent regions on SD-OCT at all time points. After 1 week, an apparent retinal tissue loss over the implant center was discernible on SD-OCT (Figure 2). The findings were well correlated with color fundus photography. Following the initial retinal thinning seen after the first week, the retina remained stable for the duration of the follow-up examinations (Figure 2; Movies S3 and S4).

On infrared cSLO, a distinct reflectivity halo appeared to surround the PET alone as well as the hRPE implants, which correlated with a loss of outer retinal SD-OCT bands (Figure 2A). The halo diameter was smaller than the original size of the bRD. PET-only implants produced a smaller halo than hRPE xenografts.

We asked whether encapsulating the graft in additional materials would help the surgical delivery and potentially reduce the retinal thinning. Temporary graft encapsulation with thermosensitive gelatin and/or plasmin-assisted vitrectomy, however, was found to cause more aggressive retinal destruction and choroidal engorgement on SD-OCT compared to unaided implantation of fetal hRPE/PET implants (Figure S2). In contrast, we found that all these negative effects were ameliorated through 1 to 2 mg intravitreal triamcinolone (TCA) injection at the end of the surgery. This long-acting synthetic corticosteroid is routinely given intraocularly to reduce immune responses. Ophthalmic and systemic complications related to the implantation procedure are summarized in Table 2.

### Characterization of hRPE Xenografts by Immunolabeling and Transmission Electron Microscopy

Animals were sacrificed and perfusion-fixed at 4 weeks posttransplantation and the recovered grafts then sectioned. Microscopic inspection revealed that the grafts had been maintained as a largely intact and continuous cell monolayer. A positive pan-cytokeratin (pCK) signal, an established RPE marker, was confirmed for fetal and adult hRPE monolayers (Figures 3A and 3C). Additional pCK reactivity was seen “underneath” the cell carrier, likely from host RPE. Moreover, fetal and adult hRPE on PET carriers stained positively for the human-specific marker SC121, confirming survival of human RPE for 1 month as a monolayer (Figure 3). Costaining of SC121 with an antibody to MCT1 and Ezrin, both apical membrane markers, further confirmed that the RPE were (still) polarized (Figure 3). SC121^+^ RPE transplants were negative for the expression of the cell-cycle marker ki67, the proliferation marker phosphohistone H3, and for the apoptotic marker caspase-3 (Figures 3F–3H), indicating absence of proliferation and apoptosis. We estimated the total human RPE cell survival to be approximately 95% after 1 month by using the SC121 positivity; we measured the total length of the carrier and the length of SC121 stain and calculated the percent coverage of SC121 over the total carrier length. On transmission electron micrography (TEM), polarized RPE cells were observed on the PET carriers from both fetal and adult transplants (Figures 3I and 3J). These results confirm survival of polarized human RPE from fetal and adult donors xenografted into rabbit SRS over 4 weeks.

### Effect of Systemic Immunosuppression on Fetal RPE Transplant and Retinal Integrity

**Figure 4:**
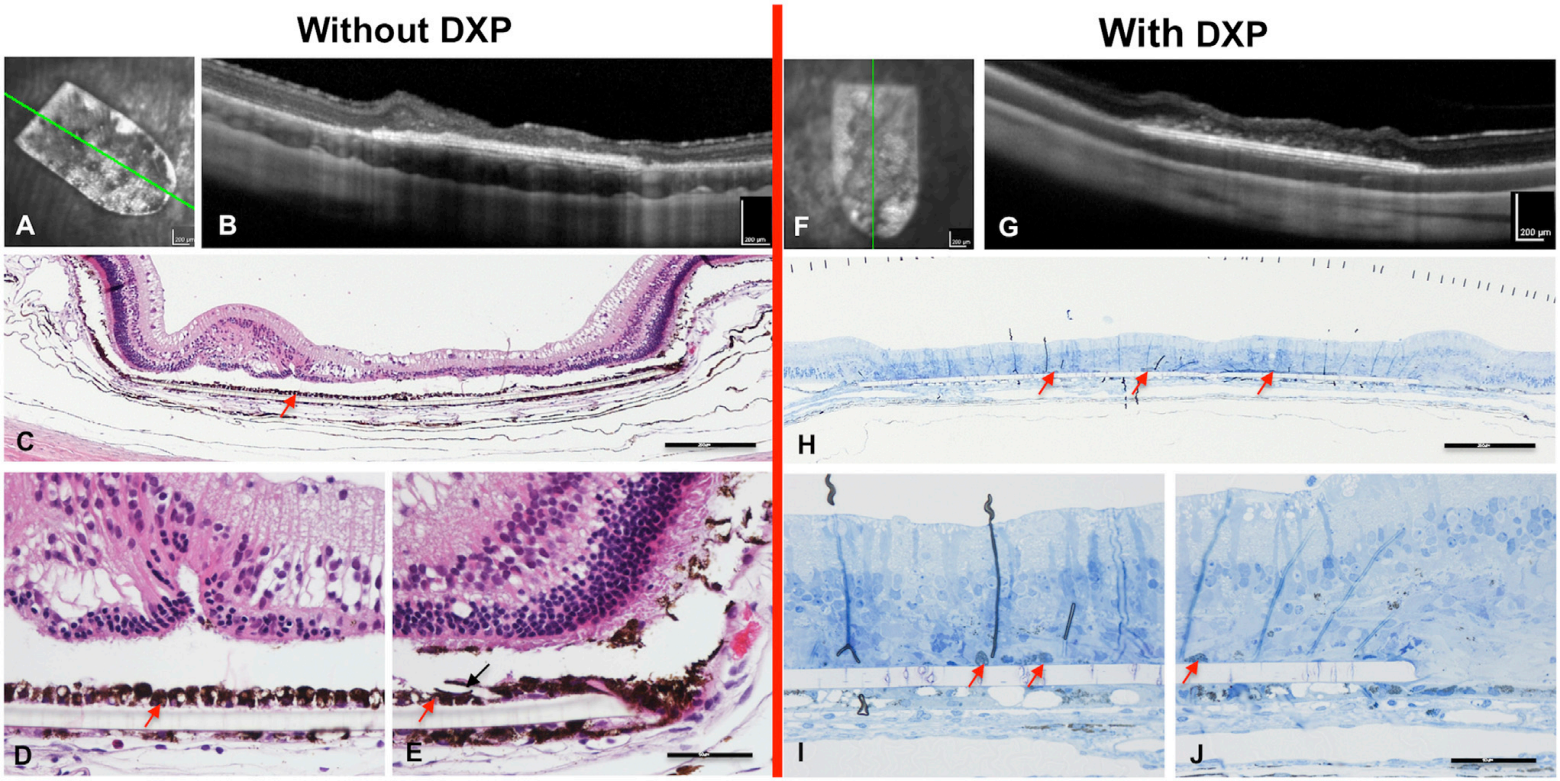
Effect of Systemic Immunosuppression on Fetal hRPE Transplant and Retinal Integrity (A–J) Immunosuppression was induced preoperatively in 14 animals with DXP. Controls and DXP-treated animals received an intravitreal injection of 1 to 2 mg TCA given at the end of the procedure. Animals were imaged at day 28 post-OP with cSLO infrared reflectance imaging (A and F), SD-OCT (B and G), and processed for histology (C–E and H–J) on the same day. Notice the rather continuous fetal hRPE layer on PET carriers without systemic DXP immunosuppression (C–E), whereas pigmented cells on DXP-suppressed samples appear mottled and discontinuous (H–J). By contrast, preservation of inner retinal reflectance layers on SD-OCT was possible in 3 of 11 survivors with DXP suppression. Red arrows in (C)–(E) and (H)–(J) point to putative hRPE transplant; black arrow (E) and retinal detachment in (C)–(E) are histologic processing artifacts. Scale bars, 200 μm (A, B, F, and G), 250 μm (C, E, and H), and 50 μm (J). See also Movie S5.

We conducted a consecutive series of transplants with preoperative, systemically immunosuppressed animals, aiming to improve preservation of the neural retina above the fetal RPE monolayer xenograft. Systemic dexamethasone (DXP) immunosuppression (2.5–3 mg/kg/day intramuscularly) in 14 rabbits resulted in inconsistent maintenance of the inner retinal layers (3 of 14), yet always leading to an atrophy of the outer photoreceptor cell layer above the hRPE implant (Figure 4; Movie S5). The surviving RPE cells appeared pleiomorphic and scattered into patches on the PET carrier. By contrast, animals that did not receive systemic immunosuppression, but were treated with TCA injection, had a complete disarray and/or absence of all retinal layers above the implant, but the hRPE monolayer was much better preserved.

Remarkably, on SD-OCT, near-normal choroidal reflectivity patterns were seen underneath the fetal hRPE implant as early as 1 week postimplantation in most (9 of 14) DXP-suppressed animals (Figure 4G), similar to areas with normal retina or PET carrier-only implants. Nonimmunosuppressed rabbits showed a distinctly diminished reflectivity and locally increased volume within the choroid underneath the fetal hRPE grafts, with no apparent histomorphologic correlate at 4 weeks postimplantation for these choroidal changes on SD-OCT. Perhaps mediated through suppression of all peripheral blood leukocytes (Jeklova et al., 2008), systemic DXP seemed to effectively dampen choroidal immune reactions underneath fetal hRPE grafts. See Table 2 for DXP-related complications.

In summary, systemic immunosuppression with DXP resulted in poor survival of fetal hRPE transplants, whereas local administration of TCA intravitreally improved surgical outcome.

## Discussion

The advances in hESC, iPSC, and RPESC technology have opened new opportunities to test whether transplantation of RPE into the SRS of patients with RPE-related diseases might restore some of the lost RPE function, ultimately leading to preservation or restoration of vision. One major unknown is whether these RPE cells can survive as a monolayer in the SRS and maintain their identity. Here, we demonstrate that polarized human fetal and adult RPE monolayers cultured on a polyester matrix can survive grafting into the rabbit SRS, thus resolving many of the current roadblocks concerning clinical RPE monolayer transplantation.

RPESCs isolated from elderly donor eyes were expanded and successfully differentiated to generate approximately 1 × 10^8^ RPE per donor. Considering 5 × 10^4^ or fewer RPE cells are required to cover the macula, using adult hRPE from one donor has the potential to treat many hundreds of patients. While promising, the expansion potential of adult hRPE is limited and the generation of multiple banks would be required to enable treatment of the millions of patients suffering from eye diseases such as AMD. One potential alternative to adult hRPE thought to have an unlimited expansion potential is hESC- and iPSC-derived RPE. However, a recent study shows changes in the physiology of iPSC-RPE after serial passaging (Singh et al., 2013); therefore, the difference between these lines and adult hRPE with regard to their expansion capabilities may not be significant.

The adult RPESC-derived RPE cultures were shipped live from the US to the German surgical team. Despite the transportation stress, the cultures soon exhibited morphology and TER values comparable to preshipment levels. Successful shipment of cultured implants between clinical-grade cell production to distant surgical sites should therefore be possible, hinting at the potential rapid spread of therapy adoption. Very little is known regarding optimal conditions for RPE implant shipment, and our studies have established conditions for successful cultured RPE monolayer transport.

Here, we show that, similar to fetal hRPE (Liao et al., 2010), adult hRPE cultures can exhibit characteristics similar to native adult human RPE. Cultured adult hRPE had TER measurements that were actually closer to native adult RPE than cultured fetal hRPE. These two RPE culture systems were developed independently in separate laboratories and as such used slightly different culture methods. More detailed comparison is warranted between human native fetal and adult tissues and RPE cultures made from these cells in order to determine the optimal cells for a particular target patient population.

After implantation into the rabbit eye, both fetal and adult RPE grown on a biocompatible, permeable polyester matrix survived and maintained key properties. Moreover, we observed no occurrence of scarring on the retinal surface (due to proliferative vitreoretinopathy) or graft proliferation. The fact that both fetal and adult hRPE behaved similarly suggests these two distinct RPE populations share comparable features with regard to transplantation.

We tested whether gelatin and plasmin could facilitate implant placement in the SRS but found survival of the RPE cells adversely affected. Gelatin and plasmin have been reported to be proinflammatory (Huang et al., 1998; Lai, 2009; Verstraeten et al., 1993), which may explain the reduced and patchy RPE coverage on PET carriers we observed in these cases at 4 weeks postimplantation (data not shown).

Remarkably, fetal and adult hRPE monolayers survived without systemic immunosuppression, which agrees with a previous fetal hRPE carrier transplant study (Bhatt et al., 1994). Our study now extends these findings to adult hRPE. Importantly, we demonstrate that adult hRPESC-derived RPE monolayers maintained cell-polarity markers up to 4 weeks postgrafting. A significant body of evidence now suggests that the RPE actively modulates the immune system via cell-surface and secretory mechanisms; that is, it has qualities of an immune-privileged tissue (Sugita, 2009). Like other epithelial cells, RPEs in single-cell suspension lose their polarized cytoskeletal and membrane-protein distribution, likely becoming more susceptible to immune rejection. Consistent with this, single-cell suspensions of RPE do not survive when transplanted underneath the kidney capsule, whereas intact RPE sheets do (Wenkel and Streilein, 2000). Suspensions of human fetal RPE transplanted into the rabbit SRS showed cellular infiltration with subsequent destruction (Gabrielian et al., 1999). In contrast, unsupported, cultured patches of fetal human RPE transplanted into albino rabbits seemed to survive 1 month, after which they showed signs of rejection (Sheng et al., 1995). Hence, it appears that maintaining RPE intercellular connectivity, i.e., as monolayers, is beneficial to prevent rejection. We therefore speculate that survival of these fetal and adult RPE xenografts in rabbits is promoted by their maintenance as a polarized monolayer. It is possible that xenograft survival in our model was also facilitated due to implant placement above an intact host RPE, because the SRS itself has also been considered an immune privileged site (Jiang et al., 1994). Destruction of the RPE by sodium iodate abolishes such immune privilege (Wenkel and Streilein, 1998). However, we note that, in the study by Bhatt et al. (1994) in which fetal hRPE cell monolayers survived 6 weeks, they removed the host RPE prior to transplantation, indicating that graft survival was not due to the host RPE layer creating an immune- privileged SRS.

We found that systemic DXP immunosuppression was less successful than local administration of TCA but view the translational value of these findings cautiously. Little is known about differences in immune rejection between RPE xenografts, as done here, and allografts as contemplated in patients. To our knowledge, there have been no direct comparisons of these two scenarios, and we note that species and animal strain differences may also be relevant. Attempts to compare the immune responses in the SRS with other xenograft models may not be appropriate because of the unique immunologic environment underneath the retina. In RPE allografts, major histocompatibility complex II expression by the RPE (and its subsequent recognition by T cells; Zhang and Bok, 1998), along with cytokine production (interleukin [IL]-6 and interferon γ) have been implicated in rejection (Enzmann et al., 2000). In contrast, RPE xenografts in rat or rabbit elicit macrophage infiltration and subsequent destruction (Carr et al., 2009; Gabrielian et al., 1999). Cytokines (IL-1 and IL-6) have also been implicated in such a reaction (Abe et al., 1999). (Thymic) lymphocyte infiltration has not been demonstrated in more than 20 published studies on RPE xenografts, yet both cyclosporine and tacrolimus, which are thought to suppress T-cell-mediated rejection, showed some efficacy in delaying xenograft rejection in rabbits (Lai et al., 2000; Wang et al., 2002). Cyclosporine has erratic plasma levels (thus requiring frequent serum measurements) and does not prevent allo- or xenograft rejection of an RPE (suspension) in rats or rabbits (Carr et al., 2009; Lai et al., 2000), thus questioning its usefulness. Tacrolimus has similar mechanisms of action but greater likelihood of toxic side effects. In an attempt to ameliorate negative retinal changes, we chose to administer DXP, as it predictably suppresses all peripheral leukocyte lineages in the rabbit (Jeklova et al., 2008). The adverse effect of DXP that we observed on xenograft survival was unexpected but perhaps mediated through the RPE glucocorticoid receptor, which promotes RPE proliferation (He et al., 1994). Taken together, no ideal, evidence-based immunosuppression protocol exists at present for hRPE xenograft models, but our data indicate that intravitreal TCA allows hRPE survival for at least 1 month, indicating that it could be valuable for longer-term studies.

Carrier implantation into the SRS with or without hRPE resulted in a degenerated retina immediately above the implant. The cause is not clear and might be multifactorial. Despite reports to the contrary (Szurman et al., 2006), we believe that the bRD is unlikely to cause retinal degeneration, because we found that producing a similar bleb without subsequent insertion of a carrier results in minimal damage to photoreceptors and negligible retinal degeneration, a finding supported by others (Ivert et al., 2002). The rabbit retina is merangiotic, meaning only part of the inner retina is supplied by retinal vessels and is therefore more dependent on the choriocapillaris for all metabolic needs compared to holangiotic species that possess a retinal vasculature that penetrates throughout the inner retina, i.e., humans and rats. Because we placed a 10-μm-thick device between the photoreceptors and the RPE, we disrupted this normally intimidate intercellular relationship with a barrier, which likely led secondarily to the observed outer retinal degeneration. Similar observations have been made using retinal chip implants, where impermeable variants of the implants result in atrophy of photoreceptor cell layers (Montezuma et al., 2006).

Porosity of cell carriers seems to play a crucial role in maintaining neural retinal health and layering, as implantation of an acellular PET carrier with 3.0 μm diameter pores results in significantly better preservation of outer retinal layers compared to a PET carrier with smaller, 0.4 μm diameter pores (B.V.S., Z.L., R.B., and F.G.H., unpublished data). However, other than a small study by Lu et al. (2012), carrier membranes with optimal porosity for retinal preservation and permissive to RPE culture were not systematically evaluated to our knowledge. hRPE on PET carriers xenografted into the SRS resulted in initial retinal edema followed by a dramatic atrophy of all overlying retinal layers within 1 week. Such a pattern was clearly different from carrier-only controls, where only the outer retinal layers degenerated. RPE-transplant-induced retinal “melting” (Del Priore et al., 2001; Rezai et al., 2000) or photoreceptor destruction (Diniz et al., 2013; Zhang and Bok, 1998) has been observed by others and remains an unsolved problem in RPE xenotransplantation (da Cruz et al., 2007). Our work indicates that, rather than surgical trauma, it is the configuration of the subretinal implants themselves which cause the observed retinal degeneration. Optimization of RPE cell carriers to better mimic physiologic Bruch’s membrane is an important subject and is currently being explored by us and many others (Liu et al., 2013; Hynes and Lavik, 2010; Kearns et al., 2012; Subrizi et al., 2012). Reducing such retinal degeneration after subretinal hRPE engraftment in rabbit SRS will improve the use of the rabbit as a cost-effective, large-eyed animal model. Future work will focus on identifying an appropriate cell carrier that more closely mimics the precious relationship between the outer retinal layer and the choriocapillaris.

## Experimental Procedures

### RPE Cultures

#### Fetal RPE

Permission to work with human RPE was obtained from the Ethics Committee of the University of Bonn. Two pairs of fetal human eyes at 19^th^ and 20^th^ week of gestation were obtained from Advanced Bioscience Resources. The tissues were transported in CO_2_-independent media (Invitrogen) supplemented with 5% normal calf serum and 100 IU penicillin/100 μg streptomycin on ice and were processed within 48 hr postenucleation. RPE cells were isolated, expanded in low calcium media, and subsequently differentiated on uncoated 10-μm-thick polyester Transwell (PET) inserts (Corning Life Sciences catalog number 3470) according to a prior protocol (Hu and Bok, 2001).

#### Adult RPE

The method for adult RPE culture followed our prior publication (Blenkinsop et al., 2013). Briefly, cadaver donor globes within 36 hr postmortem were dissected, the vitreous and retina removed, and the posterior eye-cup rinsed with calcium- and magnesium-free PBS, and then incubated in 1% dispase (2.4 IU/ml; Sigma-Aldrich catalog number D4818) for 45 min at 37°C. Sheets of RPE were gently scraped off Bruch’s membrane and then layered onto Dulbecco’s modified Eagle’s medium (DMEM)/F12 supplemented with 10% fetal bovine serum (FBS) and with 10% sucrose. After 10 min, the lower fraction containing RPE sheets was collected, centrifuged at 285 g for 5 min, resuspended in modified DMEM/F12 (see Table S1) supplemented with 15% FBS, and plated to cover roughly 50% of the well coated with placental extracellular matrix (BD Biosciences). The culture medium was changed every 2 to 3 days, with a gradual decrease in FBS from 15% down to 2% by 2 weeks. RPE sheets attached and grew to confluence within 2 weeks and were then passaged, plated into coated PET inserts (described above), and cultured for another 2 months, changing the medium two to three times weekly.

### Transepithelial Electrical Resistance Measurements

Followed standardized methods described in the Supplemental Experimental Procedures.

### Quantitative PCR

Followed standardized methods described in the Supplemental Experimental Procedures.

### Preparation of Implants

Adult hRPE cells isolated from two donors (72-year-old and 71-year-old females) cultured on PET inserts were shipped on wet ice with a commercial courier (http://www.biocair.com) from Rensselaer, NY to Bonn, Germany within 24 hr. The cultures were left to recover in the incubator at 37°C, 5% CO_2_ for 1 week until transplantation. The TER values immediately prior to implantation were around 220 and 315 Ω^∗^cm^2^ for the respective donor cultures.

Acellular and RPE-seeded implants were trephined from above PET membranes using a custom-made, bullet-shaped trephine, as described previously (Stanzel et al., 2012). RPE on the cut carriers were rinsed three times with calcium- and magnesium-containing Hank’s balanced salt solution (HBSS) immediately prior to implantation. The implant dimensions were approximately 2.2 × 1.1 mm, accommodating approximately 6–8000 cells. Prior to implantation, some membranes with and without cultured RPE were encapsulated in sterile filtered 15% porcine gelatin, bloom index 100 (Sigma-Aldrich catalog number G6144).

### Rabbit Transplantation

Sixty female chinchilla bastard rabbits weighing 2–2.5 kg (Charles River Laboratories) were utilized in experiments, which are summarized in Table 1. All procedures were approved by the state regulatory authorities of North Rhine-Westphalia (LANUV 84-02.04.2011.A130). Animals were kept in a specialized facility with temperatures between 20 and 25°C and exposure to regular daylight, in standardized individual cages with free access to food and water.

The surgical technique was further refined from our previously published protocol (Stanzel et al., 2012; Movie S1). In brief, rabbits were anesthetized by intramuscular injection of 65 mg/kg ketamine and 5 mg/kg xylazine and pupils dilated with 2.5% phenylephrine and 1% tropicamide eye drops. Eleven of 60 rabbits received an intravitreal injection of 1 U homologous rabbit plasmin in HBSS 1 hr prior to starting the procedure. Following partial surgical removal of the vitreous (two-port core-vitrectomy), a small bRD was gently raised with 25–30 μl HBSS via a 41G Teflon cannula (DORC catalog number 1270.EXT) and Hamilton syringe, thereby expanding the SRS for surgical maneuvers. Intraocular pressure was set for 30 mmHg and was consistently associated with a facile bRD induction, as compared to 20 or >50 mmHg.

The implant was then passed with the custom delivery instrument from the vitreous cavity through an enlarged incision in the retina into the SRS. RPE monolayer transplants were placed cell-carrier-side down on intact host RPE, so that the xenografted RPE faced the photoreceptors (Movie S1). Only the right eye was used for experimentation, and one implant was placed per eye.

### Immunosuppression

#### Local

Most rabbits received an intravitreal injection of 1 to 2 mg preservative-free TCA at the end of the surgical procedure (see Table 1). To control wound healing at the ocular surface, dexamethasone 1 mg/g, neomycin sulfate 3,500 IU/g, polymyxin B sulfate 6,000 IU/g ointment (Isoptomax, Alcon Pharma) was applied twice daily for 1 week postoperative onto the ocular surface.

#### Systemic

Immunosuppression was induced in 14 rabbits with intramuscular injection of DXP (Dexa-ratiopharm, Ratiopharm) over 2 days, three times/day, every 6 hr, 2.5–3 mg/kg, modified from a prior study (Jeklova et al., 2008). On the day of surgery, the animals were injected twice, 12 hr apart, with 2.5–3 mg/kg DXP, followed by a once daily maintenance dose of 2.5–3 mg/kg DXP in the morning until sacrifice. Animals were weighed regularly postimplantation and inspected by a veterinarian when necessary.

### In Vivo Follow-Up

Rabbits had repetitive noninvasive retinal imaging performed at post-OP days 4, 7, 14, and 28. Anesthesia and pupil dilation were performed as described above. The cornea was frequently lubricated with artificial tears (Optive, Allergan) to maintain its optical clarity throughout imaging.

#### Spectral Domain Optical Coherence Tomography and Confocal Scanning Laser Ophthalmoscopy

The Spectralis HRA/OCT device (Heidelberg Engineering) was used to acquire laser-interferometric reflectance OCT images of the retina and choroid, with resolutions comparable to a light microscopic section. Longitudinal and transversal line OCT scans through the center of the implant were taken with the 30-degrees-of-visual-field setting of the Spectralis. Volume scans were obtained with 60 μm distance between each scan with the device set to 20 × 20 degrees of visual field centered on the implant. To approximate human optical parameters to rabbit eyes, the Spectralis’ corneal curvature settings were set by default to 4.2 mm. Red-free and infrared cSLO images were taken at 30 degrees of visual field in the HRA mode of the device.

#### Color Fundus Photographs

A Zeiss FF 450IR camera set to 30 degrees of visual field was used to obtain color photographs to document funduscopic changes around the implant site.

### Histology

#### Perfusion Fixation and Sectioning

Rabbits were sacrificed 4 weeks after implantation in deep intramuscular anesthesia with an intracardial injection of T61. The animals were desanguinated and then perfusion-fixed with 2% glutaraldehyde (GA) or 4% formaldehyde (FA) both diluted in 0.1 M phosphate buffer. The enucleated eyes were then immersion-fixed overnight in the same fixative. Anterior segments were cut away with Vannas scissors and the eyecups photographed under a binocular microscope (Zeiss OPMI 1) with a 5-megapixel smartphone digital camera (iPhone 4, Apple). Full-thickness samples (retina-sclera) of the implantation site were cut with a surgical blade (Feather No. 22). GA probes were embedded in Spurr’s resin (Sigma, EM0300-1KT) and 1 to 2 μm semithin sections cut and then stained with toluidine blue. FA-fixed materials in paraffin were cut into 5 μm sections and stained with hematoxylin/eosin or Mayer’s hematoxylin alone (if combined with immunohistochemistry, see below). Light micrographs were taken on an Olympus BX50 microscope equipped with a Nikon DS-Vi1 digital camera.

#### Transmission Electron Microscopy

Ultrathin sections from both implant types (Spurr-embedded, see above) were analyzed using a Philips CM 10 electron microscope (Philips). Images were taken with Megaview3 CCD digital camera and coupled with digital image software analysis (Olympus).

#### Immunolabeling

##### Transwell Membranes

Transwell membranes were processed for immunocytochemistry against Claudin-19 (1:100; R&D Systems), ZO-1 (1:100; Invitrogen), Ezrin (1:100; Cell Signaling Technology), MCT-1 (1:100; Sigma), CRALBP (1:100; Abcam), RPE65 (1:100; a gift from Dr. T. Michael Redmond, National Institutes of Health [NIH]), and nuclear stain DAPI (1:10,000; Invitrogen). Secondary antibodies used at 1:1,000 dilutions were Alexa Fluor 488-goat anti-mouse IgG2A, Alexa Fluor 546-goat anti-rabbit immunoglobulin G (IgG) (H^+^L), Alexa Fluor 488-goat anti-mouse IgG1, and Alexa Fluor 546-goat anti-mouse IgG (H^+^L) (Invitrogen). Transwell membranes were fixed with ice-cold 4% FA solution for 15 min and then blocked and permeabilized with a solution containing 0.1% saponin, 5% normal goat serum, and 1% bovine serum albumin in phosphate-buffered saline. Controls included incubations of secondary antibodies in the absence of primary antibodies and block solutions in the absence of primary/secondary antibodies. Fluorescent images were collected using a Leica TCS SP5 confocal microscope.

##### Paraffin Sections

Some paraffin sections obtained from implant regions were processed for immunocytochemistry against pan-cytokeratin (1:100; catalog number ab11213, Abcam), ezrin (1:50; CST), MCT-1 (1:50; LifeSpan Biosciences), Ki67 (1:50; Sigma), phosphohistone H3 (1:50; Millipore), caspase-3 (1:50, Promega), and human-specific antibody SC121 (1:50; Stem Cells) to verify the presence of human RPE cells on the subretinally implanted cell carriers. Pan-cytokeratin was visualized with the Biotin-ExtrAvidin-AEC chromogen system. All other primary antibodies were visualized using Alexa Fluor secondary antibodies as mentioned above.

## Figures and Tables

**Table 1:** All Surgical Conditions Listed According to Implant Type, Surgical Protocol, and Use of Local and/or Systemic Immunosuppression

Implantation Protocol	Surgical Protocol^a^				Rabbits Used	Donors Used
	Gelatin	Plasmin	TCA	DXP		
RD only (19)^b^	no	no	no	no	7	
	no	no	yes	no	4	
	no	yes	yes	no	1	
	no	no	yes	yes	7	
PET alone (7)	yes	yes	yes	no	2	
	yes	yes	no	no	1	
	no	no	no	no	4	
fhRPE (40)	yes	yes	no	no	2	2^c^
	yes	yes	yes	no	5	
	yes	no	yes	no	6	
	no	no	yes	no	6	
	no	yes	yes	no	3	
	yes	no	no	no	4	
	no	no	yes	yes	14	
ahRPE (5)	no	no	yes	no	5	2^c^

Yes/no indicates variable(s) included in particular implantation protocol. TCA refers to triamcinolone acetonide, DXP is dexamethasone phosphate, RD means (bleb) retinal detachment, PET is the polyester terephthalate cell carrier, fhRPE is fetal human retinal pigment epithelium, and ahRPE is adult human retinal pigment epithelium.

a Surgeons: B.V.S. = 47, N.E. = 10, S.S. = 3.

b There were eight rabbits treated with RD only; 11 blebs created next to another implantation side.

c Cultures derived from a single donor were used for a particular implantation.

**Table 2:** List of Complications Following the Implantation Procedure

Complications	Implant Type				
	RD Only	PET Alone	ahRPE	fhRPE	
	Without DXP	Without DXP	Without DXP	Without DXP	With DXP
**Ophthalmic**					

Postoperative endophthalmitis	no	no	no	no	no

Corneal epithelial defects^a^	no	no	2/5	no	14/14
Retinal detachments, entry	3/60				
Posterior subcapsular cataracts (traumatic)	Several cases^a^				
Persistent subretinal fluid	no	no	no	2/40	

**Systemic**					

Reduction in muscle mass	no	no	no	no	14/14
Slow-healing skin abrasions at hind paws	no	no	no	no	3/14
Anesthetic complications^b^	no	no	no	no	2/14
Death rate	9/46				3/14

DXP is dexamethasone phosphate, RD means (bleb) retinal detachment, PET is the polyester terephthalate cell carrier, fhRPE is fetal human retinal pigment epithelium, and ahRPE is adult human retinal pigment epithelium.

a These cases still allowed for SD-OCT and fundus imaging.

b Malignant hypertension, tachypnea, and hyperthermia during recovery from anesthesia.

## Supplemental Information

Document S1. Supplemental Experimental Procedures, Figures S1–S3, and Table S1

Movie S1. Full Implantation Movie with Fetal hRPE/PET Implant (1) An enlarged opening in the retina (retinotomy) is created with vertical scissors. (2) The implant is inserted with a custom-made instrument from epiretinal underneath the retina. (3) The implant is pushed further into the subretinal space with half-closed branches of 23G scissors. (4) Final positioning of the graft is achieved with a 41G Teflon-cannula.

Movie S2. SD-OCT Volume Scan and Three-Dimensional Topographic Retinal Surface View of PET-Only Implant from Figure S1

Movie S3. SD-OCT Volume Scan and Three-Dimensional Topographic Retinal Surface View of Representative Fetal hRPE Implant from Figure 2

Movie S4. SD-OCT Volume Scan and Three-Dimensional Topographic Retinal Surface View of Representative Adult hRPE Implant from Figure 2

Movie S5. SD-OCT Volume Scan and Three-Dimensional Topographic Retinal Surface View of Representative Fetal hRPE Implant in DXP-Immunosuppressed Rabbit from Figure 4

## References

[bib1] Abe T., Takeda Y., Yamada K., Akaishi K., Tomita H., Sato M., Tamai M. (1999). Cytokine gene expression after subretinal transplantation. Tohoku J. Exp. Med..

[bib2] Bhatt N.S., Newsome D.A., Fenech T., Hessburg T.P., Diamond J.G., Miceli M.V., Kratz K.E., Oliver P.D. (1994). Experimental transplantation of human retinal pigment epithelial cells on collagen substrates. Am. J. Ophthalmol..

[bib3] Binder S., Stanzel B.V., Krebs I., Glittenberg C. (2007). Transplantation of the RPE in AMD. Prog. Retin. Eye Res..

[bib4] Blenkinsop T.A., Salero E., Stern J.H., Temple S. (2013). The culture and maintenance of functional retinal pigment epithelial monolayers from adult human eye. Methods Mol. Biol..

[bib5] Bonilha V.L., Finnemann S.C., Rodriguez-Boulan E. (1999). Ezrin promotes morphogenesis of apical microvilli and basal infoldings in retinal pigment epithelium. J. Cell Biol..

[bib6] Bunt-Milam A.H., Saari J.C. (1983). Immunocytochemical localization of two retinoid-binding proteins in vertebrate retina. J. Cell Biol..

[bib7] Burke J.M. (2008). Epithelial phenotype and the RPE: is the answer blowing in the Wnt?. Prog. Retin. Eye Res..

[bib8] Carr A.J., Vugler A.A., Hikita S.T., Lawrence J.M., Gias C., Chen L.L., Buchholz D.E., Ahmado A., Semo M., Smart M.J. (2009). Protective effects of human iPS-derived retinal pigment epithelium cell transplantation in the retinal dystrophic rat. PLoS ONE.

[bib9] da Cruz L., Chen F.K., Ahmado A., Greenwood J., Coffey P. (2007). RPE transplantation and its role in retinal disease. Prog. Retin. Eye Res..

[bib10] Del Priore L.V., Kaplan H.J., Tezel T.H., Hayashi N., Berger A.S., Green W.R. (2001). Retinal pigment epithelial cell transplantation after subfoveal membranectomy in age-related macular degeneration: clinicopathologic correlation. Am. J. Ophthalmol..

[bib11] Diniz B., Thomas P., Thomas B., Ribeiro R., Hu Y., Brant R., Ahuja A., Zhu D., Liu L., Koss M. (2013). Subretinal implantation of retinal pigment epithelial cells derived from human embryonic stem cells: improved survival when implanted as a monolayer. Invest. Ophthalmol. Vis. Sci..

[bib12] Enzmann V., Faude F., Wiedemann P., Kohen L. (2000). The local and systemic secretion of the pro-inflammatory cytokine interleukin-6 after transplantation of retinal pigment epithelium cells in a rabbit model. Curr. Eye Res..

[bib13] Gabrielian K., Oganesian A., Patel S.C., Verp M.S., Ernest J.T. (1999). Cellular response in rabbit eyes after human fetal RPE cell transplantation. Graefes Arch. Clin. Exp. Ophthalmol..

[bib14] He S., Wang H.M., Ye J., Ogden T.E., Ryan S.J., Hinton D.R. (1994). Dexamethasone induced proliferation of cultured retinal pigment epithelial cells. Curr. Eye Res..

[bib15] Hirami Y., Osakada F., Takahashi K., Okita K., Yamanaka S., Ikeda H., Yoshimura N., Takahashi M. (2009). Generation of retinal cells from mouse and human induced pluripotent stem cells. Neurosci. Lett..

[bib16] Hu J., Bok D. (2001). A cell culture medium that supports the differentiation of human retinal pigment epithelium into functionally polarized monolayers. Mol. Vis..

[bib17] Huang J.C., Ishida M., Hersh P., Sugino I.K., Zarbin M.A. (1998). Preparation and transplantation of photoreceptor sheets. Curr. Eye Res..

[bib18] Hynes S.R., Lavik E.B. (2010). A tissue-engineered approach towards retinal repair: scaffolds for cell transplantation to the subretinal space. Graefes Arch. Clin. Exp. Ophthalmol..

[bib19] Ivert L., Kjeldbye H., Gouras P. (2002). Long-term effects of short-term retinal bleb detachments in rabbits. Graefes Arch. Clin. Exp. Ophthalmol..

[bib20] Jeklova E., Leva L., Jaglic Z., Faldyna M. (2008). Dexamethasone-induced immunosuppression: a rabbit model. Vet. Immunol. Immunopathol..

[bib21] Jiang L.Q., Jorquera M., Streilein J.W. (1994). Immunologic consequences of intraocular implantation of retinal pigment epithelial allografts. Exp. Eye Res..

[bib22] Kearns V., Mistry A., Mason S., Krishna Y., Sheridan C., Short R., Williams R.L. (2012). Plasma polymer coatings to aid retinal pigment epithelial growth for transplantation in the treatment of age related macular degeneration. J. Mater. Sci. Mater. Med..

[bib23] Klimanskaya I., Hipp J., Rezai K.A., West M., Atala A., Lanza R. (2004). Derivation and comparative assessment of retinal pigment epithelium from human embryonic stem cells using transcriptomics. Cloning Stem Cells.

[bib24] Lai J.Y. (2009). The role of bloom index of gelatin on the interaction with retinal pigment epithelial cells. Int. J. Mol. Sci..

[bib25] Lai C.C., Gouras P., Doi K., Tsang S.H., Goff S.P., Ashton P. (2000). Local immunosuppression prolongs survival of RPE xenografts labeled by retroviral gene transfer. Invest. Ophthalmol. Vis. Sci..

[bib26] Liao J.L., Yu J., Huang K., Hu J., Diemer T., Ma Z., Dvash T., Yang X.J., Travis G.H., Williams D.S. (2010). Molecular signature of primary retinal pigment epithelium and stem-cell-derived RPE cells. Hum. Mol. Genet..

[bib27] Lim L.S., Mitchell P., Seddon J.M., Holz F.G., Wong T.Y. (2012). Age-related macular degeneration. Lancet.

[bib28] Liu Z., Yu N., Holz F., Yang F., Stanzel B. (2013). Engineering a biocompatible cell carrier with nanofeatured topography for retinal pigment epithelium transplantation. Invest. Ophthalmol. Vis. Sci..

[bib29] Lu B., Zhu D., Hinton D., Humayun M.S., Tai Y.C. (2012). Mesh-supported submicron parylene-C membranes for culturing retinal pigment epithelial cells. Biomed. Microdevices.

[bib30] Montezuma S.R., Loewenstein J., Scholz C., Rizzo J.F. (2006). Biocompatibility of materials implanted into the subretinal space of Yucatan pigs. Invest. Ophthalmol. Vis. Sci..

[bib31] Nicolini J., Kiilgaard J.F., Wiencke A.K., Heegaard S., Scherfig E., Prause J.U., la Cour M. (2000). The anterior lens capsule used as support material in RPE cell-transplantation. Acta Ophthalmol. Scand..

[bib32] Peng S., Rao V.S., Adelman R.A., Rizzolo L.J. (2011). Claudin-19 and the barrier properties of the human retinal pigment epithelium. Invest. Ophthalmol. Vis. Sci..

[bib33] Philp N.J., Yoon H., Grollman E.F. (1998). Monocarboxylate transporter MCT1 is located in the apical membrane and MCT3 in the basal membrane of rat RPE. Am. J. Physiol..

[bib34] Redmond T.M., Yu S., Lee E., Bok D., Hamasaki D., Chen N., Goletz P., Ma J.X., Crouch R.K., Pfeifer K. (1998). Rpe65 is necessary for production of 11-cis-vitamin A in the retinal visual cycle. Nat. Genet..

[bib35] Rezai K.A., Farrokh-Siar L., Godowski K., Patel S.C., Ernest J.T. (2000). A model for xenogenic immune response. Graefes Arch. Clin. Exp. Ophthalmol..

[bib36] Salero E., Blenkinsop T.A., Corneo B., Harris A., Rabin D., Stern J.H., Temple S. (2012). Adult human RPE can be activated into a multipotent stem cell that produces mesenchymal derivatives. Cell Stem Cell.

[bib37] Schwartz S.D., Hubschman J.P., Heilwell G., Franco-Cardenas V., Pan C.K., Ostrick R.M., Mickunas E., Gay R., Klimanskaya I., Lanza R. (2012). Embryonic stem cell trials for macular degeneration: a preliminary report. Lancet.

[bib38] Sheng Y., Gouras P., Cao H., Berglin L., Kjeldbye H., Lopez R., Rosskothen H. (1995). Patch transplants of human fetal retinal pigment epithelium in rabbit and monkey retina. Invest. Ophthalmol. Vis. Sci..

[bib39] Singer M.A., Awh C.C., Sadda S., Freeman W.R., Antoszyk A.N., Wong P., Tuomi L. (2012). HORIZON: an open-label extension trial of ranibizumab for choroidal neovascularization secondary to age-related macular degeneration. Ophthalmology.

[bib40] Singh R., Phillips M.J., Kuai D., Meyer J., Martin J.M., Smith M.A., Perez E.T., Shen W., Wallace K.A., Capowski E.E. (2013). Functional analysis of serially expanded human iPS cell-derived RPE cultures. Invest. Ophthalmol. Vis. Sci..

[bib41] Stanzel B.V., Holz F.G., Spaeth G.L., Kampik A. (2012). Surgical strategies for AMD. Ophthalmic Surgery: Principles and Practice.

[bib42] Stanzel B.V., Liu Z., Brinken R., Braun N., Holz F.G., Eter N. (2012). Subretinal delivery of ultrathin rigid-elastic cell carriers using a metallic shooter instrument and biodegradable hydrogel encapsulation. Invest. Ophthalmol. Vis. Sci..

[bib43] Strauss O. (2005). The retinal pigment epithelium in visual function. Physiol. Rev..

[bib44] Subrizi A., Hiidenmaa H., Ilmarinen T., Nymark S., Dubruel P., Uusitalo H., Yliperttula M., Urtti A., Skottman H. (2012). Generation of hESC-derived retinal pigment epithelium on biopolymer coated polyimide membranes. Biomaterials.

[bib45] Sugino I.K., Sun Q., Wang J., Nunes C.F., Cheewatrakoolpong N., Rapista A., Johnson A.C., Malcuit C., Klimanskaya I., Lanza R., Zarbin M.A. (2011). Comparison of FRPE and human embryonic stem cell-derived RPE behavior on aged human Bruch’s membrane. Invest. Ophthalmol. Vis. Sci..

[bib46] Sugita S. (2009). Role of ocular pigment epithelial cells in immune privilege. Arch. Immunol. Ther. Exp. (Warsz.).

[bib47] Szurman P., Roters S., Grisanti S., Aisenbrey S., Schraermeyer U., Lüke M., Bartz-Schmidt K.U., Thumann G. (2006). Ultrastructural changes after artificial retinal detachment with modified retinal adhesion. Invest. Ophthalmol. Vis. Sci..

[bib48] van Zeeburg E.J., Maaijwee K.J., Missotten T.O., Heimann H., van Meurs J.C. (2012). A free retinal pigment epithelium-choroid graft in patients with exudative age-related macular degeneration: results up to 7 years. Am J Ophthalmol.

[bib49] Verstraeten T.C., Chapman C., Hartzer M., Winkler B.S., Trese M.T., Williams G.A. (1993). Pharmacologic induction of posterior vitreous detachment in the rabbit. Arch. Ophthalmol..

[bib50] Wang F., Xu P., Wu J.H., Xia X., Sun H.L., Xu X., Huang Q. (2002). [Long-term outcome of gfp gene modified human RPE xenografts into the subretinal space of rabbits]. Sheng Wu Hua Xue Yu Sheng Wu Wu Li Xue Bao (Shanghai).

[bib51] Wenkel H., Streilein J.W. (1998). Analysis of immune deviation elicited by antigens injected into the subretinal space. Invest. Ophthalmol. Vis. Sci..

[bib52] Wenkel H., Streilein J.W. (2000). Evidence that retinal pigment epithelium functions as an immune-privileged tissue. Invest. Ophthalmol. Vis. Sci..

[bib53] Zhang X., Bok D. (1998). Transplantation of retinal pigment epithelial cells and immune response in the subretinal space. Invest. Ophthalmol. Vis. Sci..

