# Supplementary material for: Human RPE Stem Cells Grown into Polarized RPE Monolayers on a Polyester Matrix Are Maintained after Grafting into Rabbit Subretinal Space
Source: Stem Cell Reports. 2014 Jan 2;2(1):64–77. doi: 10.1016/j.stemcr.2013.11.005 (PMC3916756; doi:10.1016/j.stemcr.2013.11.005)
Supplement: Document S1. Supplemental Experimental Procedures, Figures S1–S3, and Table S1 [file mmc1.pdf]

Stem Cell Reports, Volume *2*

Supplemental Information

Human RPE Stem Cells Grown into Polarized RPE Monolayers on a Polyester Matrix Are Maintained after Grafting into Rabbit Subretinal Space

Boris V. Stanzel, Zengping Liu, Sudawadee Somboonthanakij, Warapat Wongsawad, Ralf Brinken, Nicole Eter, Barbara Corneo, Frank G. Holz, Sally Temple, Jeffrey H. Stern, and Timothy A. Blenkinsop

Inventory of Supplemental Information

Main Figure 1 Supplemental Information

Figure S1
Transverse images from Z-stack taken from the apical and basolateral regions of the RPE

Main Figure 2 Supplemental Information

Figure S2:
Sham surgery controls: Bleb retinal detachment with and without implantation of PET only.

Figure S3:
Adverse effect of plasmin & gelatin on subretinal implantation of fetal hRPE monolayer.

Movie S1:
Full implantation video with fetal hRPE/PET implant.

Movie S2:
SD-OCT volume scan and 3D topographic retinal surface view of PET only implant from Fig. S1

Movie S3:
SD-OCT volume scan and 3D topographic retinal surface view of representative fetal hRPE implant from Figure 2

Movie S4:
SD-OCT volume scan and 3D topographic retinal surface view of representative adult hRPE implant from Figure 2

Main Figure 4 Supplemental Information

Movie S5:
SD-OCT volume scan and 3D topographic retinal surface view of representative fetal hRPE implant in DXP-immunosuppressed rabbit from Figure 4

Supplemental Table S1:
Adult hRPE basal media formulation with 2% serum.

Supplemental Experimental Procedure S1:
Transepithelial resistance measurements

Supplemental Experimental Procedure S2:
Quantitative PCR of adult hRPE

Supplemental Figures and Legends, Tables, Experimental Procedures

Supplemental Figures

Supplemental Figure S1

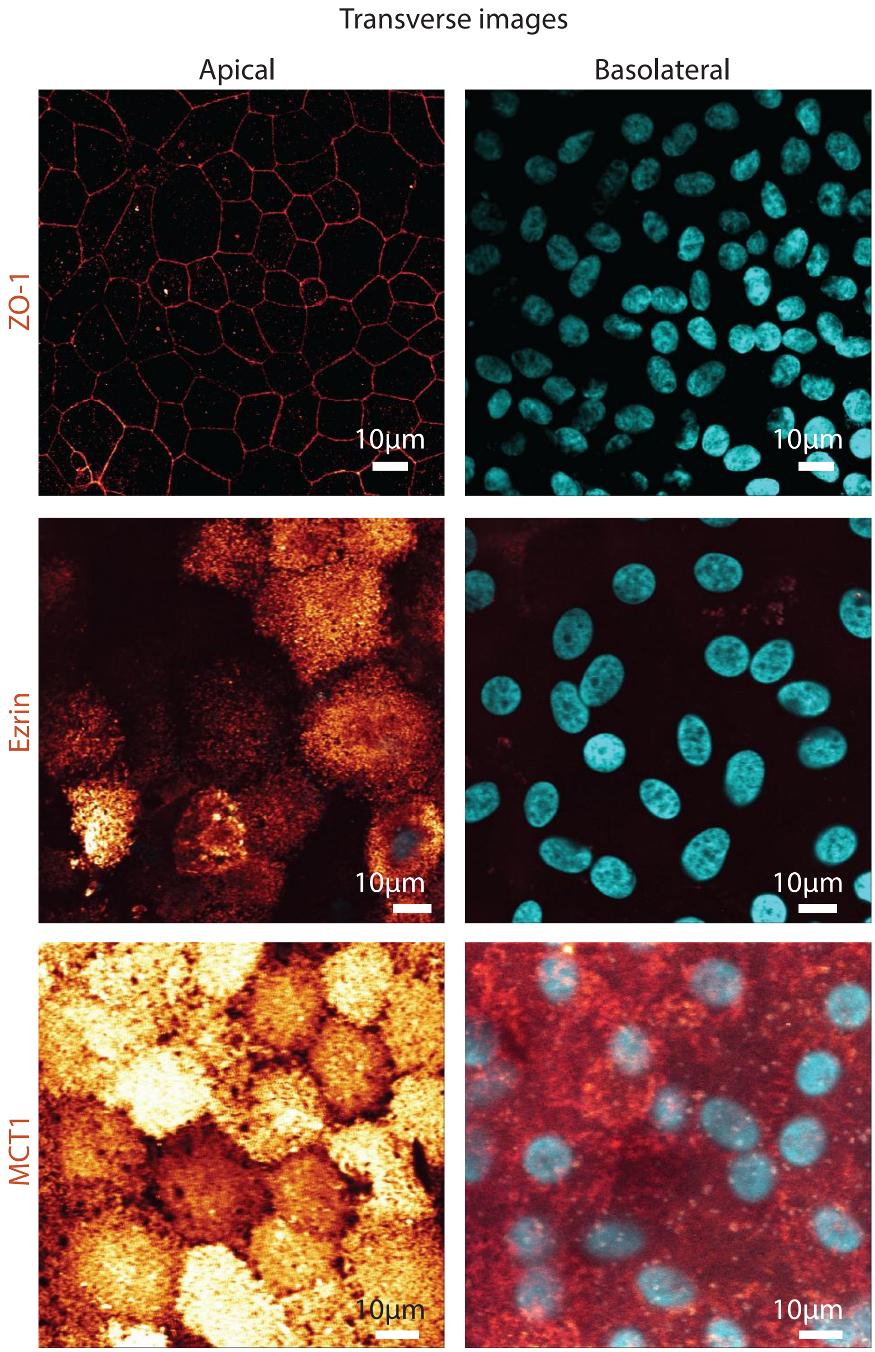

Supplemental Figure S2

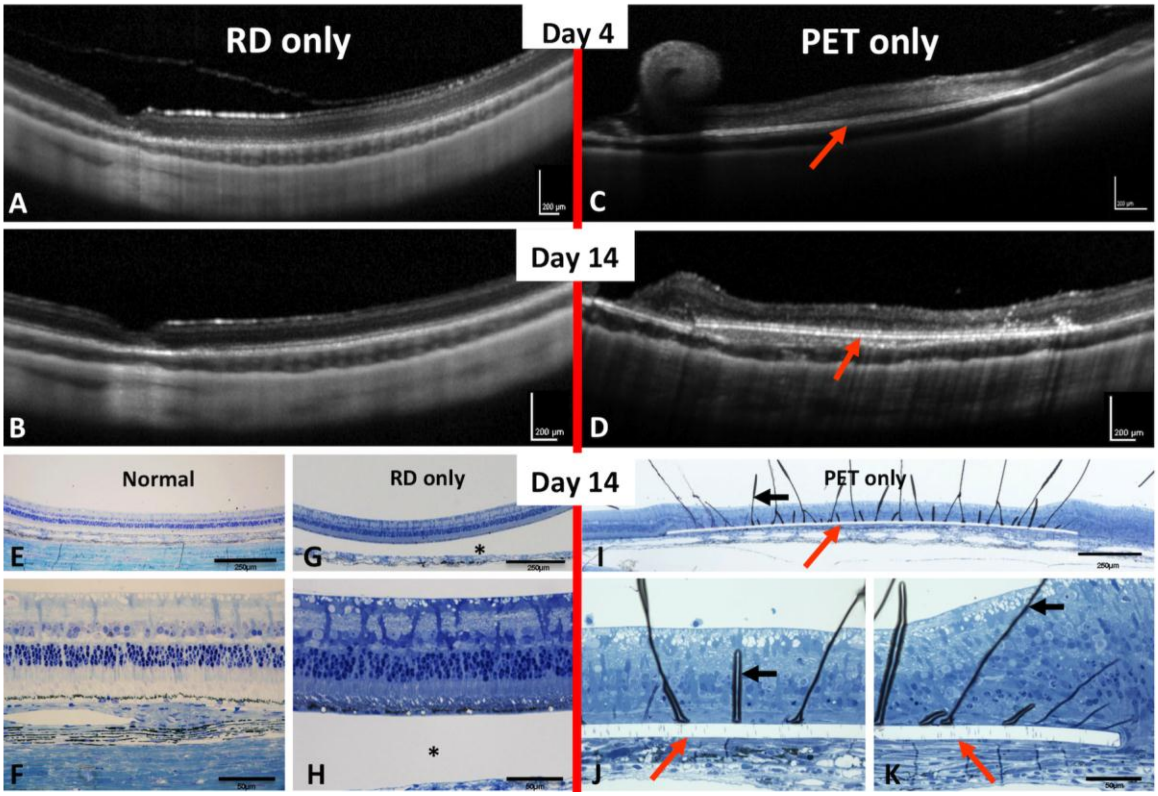

Supplemental Figure S3

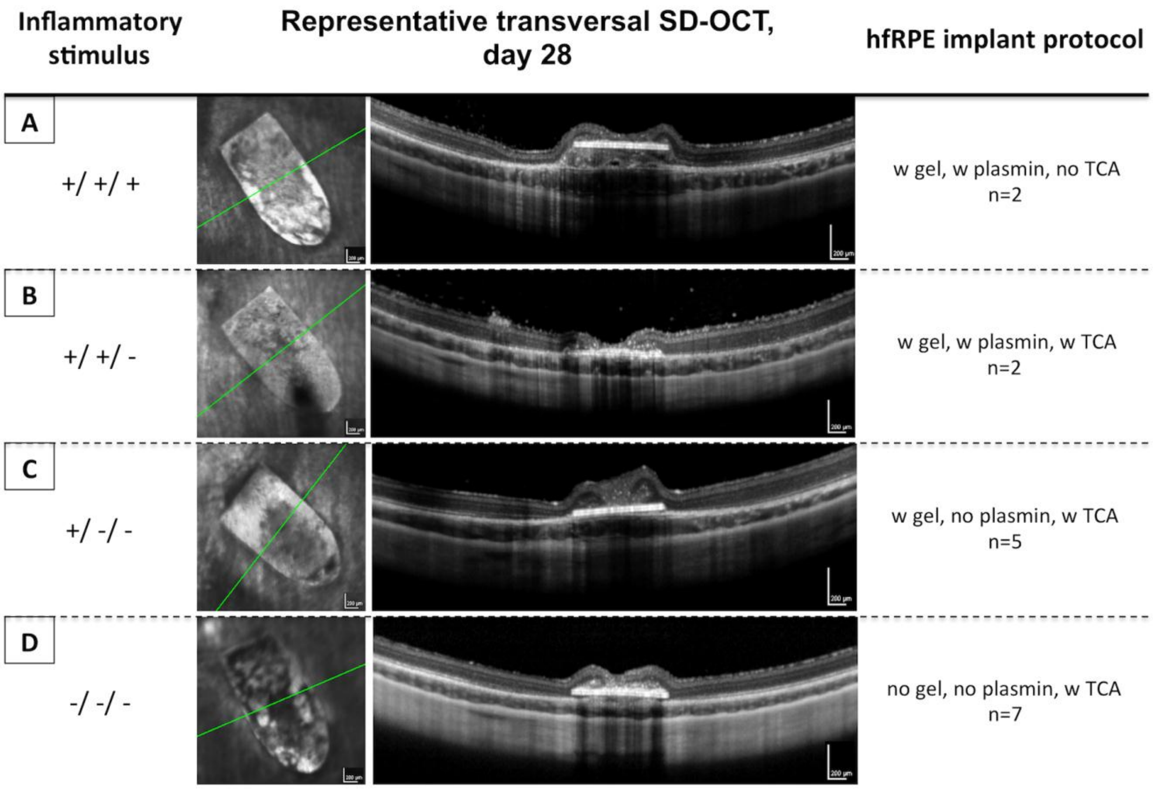

Supplemental Legends

Figure S1:

Transverse images from Z-stack taken from the apical and basolateral regions of the RPE. Apical images contain the associated immunofluorescence, whereas basolateral images show either none or relatively negligible levels of polarized RPE marker signal.

Figure S2:

Sham surgery controls: Bleb retinal detachment with and without implantation of PET only.

(A, C) The neural retina overlying the bleb re-attached within 3-4 days. (A, B, G, H) Controls with bleb RDs only, showed near normal retinal layering on OCT and histology. Compared to normal rabbit eyes though, RD only (E, F vs. G, H) seems to induce some degree of Müller cell hypertrophy along with slight irregularities in photoreceptor outer segment alignment. Asterisk in (G, H) indicates RPE/ choroid separation, a post-mortem processing artifact. (C, D, I- K) The area around the PET-only implant without cells (red arrow) showed significant subretinal scarring with atrophy of outer retinal layers, but retained, seemingly continuous inner retinal layers. No inflammatory, except for hypertrophied RPE cells were observed around PET in semithin sections. Black arrows point to processing artifacts, i.e. resin stretch marks during sectioning. Scale bars in (A-D) indicate 200µm, while in (E, G& I) they represent 250µm, and in (F, H& K) they are 50µm; (J & K) are taken at the same magnification. Histologic processing was carried out immediately after last OCT imaging, at day 14 postoperative. See also Movie S4.

Figure S3:

Adverse effect of plasmin & gelatin on subretinal implantation of fetal hRPE monolayer.

(A) Implantation aided with 1IU intravitreal plasmin 1h prior implantation and gelatin (gel) encapsulation of the fetal hRPE without triamcinolone (TCA) resulted in significant sub-implant choroidal engorgement and subretinal scarring. This stimulus was classified most pro-inflammatory (+/+/+). (B) Choroidal and subretinal reactions observed in the former protocol were ameliorated with an intravitreal injection of 1-2mg TCA at the end of the procedure, thereby reducing inflammation to (+/+/-). (C) When the implantation was conducted with gelatin-encapsulation and TCA but without plasmin-pretreatment, the inflammatory stimulus was presumed to be even less (+/-/-), thereby apparently improving preservation of the neural retina on central implant transverse OCT scans. (D) Omitting all surgical implantation aids (i.e. no gelatin and no plasmin), but injecting TCA at the end of the procedure resulted in most preserved neural retinal tissue at the standardized central implant location presumably as a consequence of least inflammatory stimulus (-/-/-). n in right column corresponds with animals that completed follow up according to Table 1. All scale bars represent 200µm.

Movie S1:

Full implantation movie with fetal hRPE/PET implant.

1) An enlarged opening in the retina (retinotomy) is created with vertical scissors. 2) The implant is inserted with a custom-made instrument from epiretinal underneath the retina. 3) The implant is pushed further into the subretinal space with half-closed branches of 23G scissors. 4) Final positioning of the graft is achieved with a 41G Teflon-cannula.

Movie S2:

SD-OCT volume scan and 3D topographic retinal surface view of PET only implant from Fig. S1

Movie S3:

SD-OCT volume scan and 3D topographic retinal surface view of representative fetal hRPE implant from Figure 2

Movie S4:

SD-OCT volume scan and 3D topographic retinal surface view of representative adult hRPE implant from Figure 2

Movie S5:

SD-OCT volume scan and 3D topographic retinal surface view of representative fetal hRPE implant in DXP-immunosuppressed rabbit from Figure 4

Supplemental Table S1:

Adult hRPE basal media formulation with 2% serum.

Basic RPE Media Formulation (total 300 ml)		
Component	Volume	Source
DMEM/F12, High Glucose	260.5 ml	Invitrogen, cat. no. 11960-077
Penicillin/Streptomycin 1%	3 ml	Invitrogen, cat. no. 15140-122
N1 Supplement	1.5 ml	Sigma, N-6530
Fetal Bovine Serum	15 ml	GIBCO-Invitrogen, cat. no. 10082147
Non-essential amino acids (10mM)	3 ml	GIBCO-Invitrogen, cat. no. 11140
L-glutamine 2mM	3 ml	GIBCO-Invitrogen, cat. no. 25030081
Taurine 75 mg		(Sigma, cat. no. T-0625)
Hydrocortisone 6 µg		Sigma, cat. no. H-0396
Triiodo-thyronin 0.0039 µg		Sigma, cat. no. T-5516

Supplemental Experimental Procedures

Supplemental Experimental Procedure S1:

Transepithelial resistance measurements

Transepithelial electrical resistance (TER) of RPE monolayers was measured using the Millicell ERS voltohmmeter (Millipore Corporation, Billerica, MA, USA), according to modified manufacturer's instructions. In brief, the cultures were equilibrated outside the incubator at room temperature for 20 minutes before the experiment. Measurements were performed in (unchanged) maintenance culture media and repeated three times at three different positions of each well and averages used for further analysis. The background resistance was determined in analogy from a blank culture insert bathed in the same media (without cells) at each experiment and subtracted from the respective experimental condition. The measurements are reported as resistance in ohms times the area in square centimeter [Ω*cm^2].

Supplemental Experimental Procedure S2:

Quantitative PCR of adult hRPE

RNA isolation was performed by following the protocol included in the RNAeasy plus Mini Kit (Qiagen). The relative differences (n-fold) in gene expression of a characteristic marker were determined by using cyclophilin G as an internal control (housekeeping gene) for each reaction set. Each sample was conducted in triplicate. Data is represented as the mean relative change ± SEM of the n separate qPCR amplification reactions. The primer sequences, annealing temperatures, and product size are shown below.

Three step qPCR cycling conditions for SYBR Green (Invitrogen) I assay			
**Step**	**Denature**	**Anneal**	**Extend**

**number**			
1 cycle	95 ^{o}C for 10 min		
40 cycles	95 ^{o}C for 15 sec	60 ^{o}C for 1 min	72 ^{o}C for 45 sec
Dissociation step	95°C, 15 sec<br>60°C, 1 min<br>95°C, 15 sec<br>60°C, 1 min		

List of primers used for Real Time PCR on adult human RPE

Human Gene	Forward 5'-3'	Reverse 3'-5'	Product Size (bp)	T_{ann} (^{o}C)	Gene Bank ID
MITF	TTGTCCATCTGCCTCTGAGTAG	CCTATGTATGACCAGGTTGCTTG	87	55	NM_198178
OTX2	CCATGACCTATACTCAGGCTTCAGG	GAAGCTCCATATCCCTGGGTGGAAAG	211	62	NM_021728
BEST1 (BESTROPHIN)	CCTTTATGGGCTCCACCTTCAACATC	CAGTAGTTTGGTCCTTGAGTTTGCCC	166	65	NM_004183
TYR	TTGGCAGATTGTCTGTAGCC	AGGCATTGTGCATGCTGCTT	78	60	NM_340037
TRYP1	TGCTCCAGACAACCTGGGATACA	TGCAACCAGTACAAAGCGCCAAC	108	60	NM_000550
TRYP2	ACTGGTGGCTTTGGTTGGTCTT	TTGGCCAGCCTCTTCTCTTAGGT	160	60	NM_001922
Cyclophilin G	CTTGTCAATGGCCAACAGAGG	GCCCATCTAAATGAGGAGTTGGT	82	60	NM_004792
